# Immunogenicity evaluation of primary polio vaccination schedule with inactivated poliovirus vaccines and bivalent oral poliovirus vaccine

**DOI:** 10.1186/s12879-024-09389-8

**Published:** 2024-05-28

**Authors:** Jiawei Xu, Yang Liu, Wei Qiu, Wenwen Li, Xiaoxiao Hu, Xia Li, Qiang Fan, Wenge Tang, Yujie Wang, Qing Wang, Ning Yao

**Affiliations:** 1 EPI Department, Chongqing Municipal Center for Disease Control and Prevention, No.8 Changjiang 2nd Street, Yuzhong District, Chongqing, 400042 China; 2 EPI Department, Hechuan District Center Disease Control and Prevention, Chongqing, China; 3 EPI Department, Liangping District Center Disease Control and Prevention, Chongqing, China; 4 EPI Department, Rongchang District Center Disease Control and Prevention, Chongqing, China; 5 EPI Department, Zhongxian County Center Disease Control and Prevention, Chongqing, China; 6 School of Public Health, Chongqing Medical University, Chongqing, China; 7 Department of Health Statistics, College of Preventive Medicine, Army Medical University, Chongqing, 400038 China

**Keywords:** Immunogenicity, IPV, bOPV, Sequential immunization

## Abstract

**Background:**

To assess the immunogenicity of the current primary polio vaccination schedule in China and compare it with alternative schedules using Sabin or Salk-strain IPV (sIPV, wIPV).

**Methods:**

A cross-sectional investigation was conducted at four sites in Chongqing, China, healthy infants aged 60–89 days were conveniently recruited and divided into four groups according to their received primary polio vaccination schedules (2sIPV + bOPV, 2wIPV + bOPV, 3sIPV, and 3wIPV). The sero-protection and neutralizing antibody titers against poliovirus serotypes (type 1, 2, and 3) were compared after the last dose.

**Results:**

There were 408 infants completed the protocol. The observed seropositivity was more than 96% against poliovirus types 1, 2, and 3 in all groups. IPV-only groups induced higher antibody titers(GMT) against poliovirus type 2 (Median:192, QR: 96–384, *P*<0.05) than the “2IPV + bOPV” group. While the “2IPV + bOPV” group induced significantly higher antibody titers against poliovirus type 1 (Median:2048, QR: 768–2048, *P*<0.05)and type 3 (Median:2048, QR: 512–2048, *P*<0.05) than the IPV-only group.

**Conclusions:**

Our findings have proved that the two doses of IPV with one dose of bOPV is currently the best polio routine immunization schedule in China.

## Background

Poliomyelitis (polio) is a highly contagious disease caused by one of three poliovirus serotypes (poliovirus types 1, 2, and 3) [1].In 1988, the World Health Assembly adopted a resolution for the worldwide eradication of polio. Since the strategies for polio eradication are fully implemented, wild poliovirus (WPV) cases have decreased by over 99%, from an estimated case of 350,000 in 1988 to 175 reported cases in 2019. The type 2 WPV (WPV2) was eradicated in 1999 and no case of type 3 WPV (WPV3) has been found since the last reported case in Nigeria in November, 2012. Both the two strains have been officially certified as globally eradicated [2]. The Polio Eradication and Endgame Strategic Plan 2013–2018 has called for globally synchronized sequential removal of Sabin virus strains contained in trivalent oral attenuated polio vaccine (tOPV), beginning with poliovirus type 2 [3].

In 2016, there was a globally synchronized “switch” to replace tOPV with bivalent oral poliovirus vaccine (bOPV) containing only poliovirus types 1 and 3 [4, 5]. The coordinated withdrawal was preceded by the introduction of one dose of inactivated polio vaccine (IPV) into national immunization schedules for intensified efforts to increase type 2 population immunity, but was not successfully or timely executed in many countries. After the emergence of circulating vaccine-derived poliovirus (cVDPV) type 2 in 2019, waning type 2 mucosal immunity and gaps in immunization activities have resulted in the spreading of cVDPV2, and Afghanistan and Pakistan are now experiencing the co-circulation of type 1 WPV (WPV1) and cVDPV2. Inadequate response to new outbreak detections, delayed immunization campaigns, and poor coverage with monovalent type 2 oral poliovirus vaccine (mOPV2) have contributed to wide and persistent transmission of cVPDV2 in countries with initial outbreaks, caused importations into neighborhoods and seeding of new cVDPV2 lineages [6–11]. A total of 154 cVDPV2 outbreaks from 82 cVDPV2 emergences have been reported in 48 countries, of which, seventeen (35%) countries experienced their first cVDPV emergences and outbreaks in 2021 (eight) and 2022 (nine) since the switch [12, 13].

Because of cultural and economic ties and large-scale cross-border population movement, the world continues to face threats of WPV1 and cVDPV2. Since the implementation of the polio immunization strategy of 1 dose of IPV followed by 3 doses of bOPV in 2016, China has adjusted its routine polio immunization strategy of two IPV doses at age 2 and 3 months followed by two bOPV at 4 months and 4 years of age in 2020. According to Chinese Instructions for Children with National Immunization Program Vaccines, the children and their guardians can choose to receive non-immunization program vaccines containing polio components as an alternative to routine immunization schedule vaccines for polio. This means that 2 doses of IPV followed by 1 dose of bOPV and 3 doses of IPV are both considered to be completion of the primary immunization against polio in China.

Salk strain IPV (wIPV) was first introduced into routine immunization schedule for children in China in 2016 and is currently available as containing WPV strains, and our self-developed IPV derived from Sabin strain IPV(sIPV) is also used in the current polio vaccine sequential immunization schedule since 2016. Though the sero-positivites for three poliovirus serotypes after routine primary poliovirus vaccination were high from 74.58–100% [14–16] in China, the antibodies titers for three poliovirus serotypes were quite different. So, it is important for Chinese policymakers to know the efficacy of sequential immunization using sIPV/wIPV with bOPV and the number of doses of IPV needed for adequate type 2 poliovirus protection in current situation [17]. To provide scientific evidence for the conversion of polio vaccination procedures, we assessed the immunogenicity of the routine primary poliovirus vaccination series (s/wIPV-s/wIPV-bOPV) and the alternative series (s/wIPV-s/wIPV-s/wIPV) in China and compared the immunogenicity of sequential vaccination with different strains of polio vaccines.

## Methods

### Study design and subjects

The cross-sectional study was conducted at four sites (Liangping District, Hechuan District, Rongchang District and Zhongxian County) from May 2021 to April 2022 in Chongqing, China. All infants who came for their first polio vaccination in the vaccination clinic in the four sites were invited to participate in our study. Eligible participants were full-term infants aged 60–89 days, weighted more than 2.5 kg at birth, were healthy on physical examination with no obvious medical conditions, and had no contraindications to vaccination. Infants who were immunodeficient or had taken immunosuppression drugs during the last 2 months, or had contraindications to polio vaccine were excluded from our study. For the free routine poliovirus vaccination schedule, the infants were administered two IPV and one bOPV dose at 2, 3, and 4 months of age; and parents were allowed to choose three doses of IPV schedule as an alternative, with the third dose of IPV paid for vaccination by self. The use of sIPV and wIPV depended on vaccine availability in local clinic. Then subjects were divided into four groups by different primary vaccination schedules: wIPV-wIPV-wIPV(3wIPV), sIPV-sIPV-sIPV(3sIPV), wIPV-wIPV-bOPV(2wIPV + bOPV) and sIPV-snIPV-bOPV (2sIPV + bOPV).

The sample size was calculated based on the equation below with an assumed seroprotection rate of 95%,

$$n = (\frac{{z_{{\alpha \mathord{\left/{\vphantom {\alpha 2}} \right.\kern-\nulldelimiterspace} 2}}^2 \times p \times \left( {1 - p} \right)}}{{{\delta ^2}}}) \times deff$$

${z_{{\alpha \mathord{\left/{\vphantom {\alpha 2}} \right.\kern-\nulldelimiterspace} 2}}}$= 1.96, δ= 0.03 was the maximum allowed error. The $deff$ was the design effect (2), which resulted in a final sample size of 406.

Questionnaires were used to collect participants’ demographic information (gender, age, region, vaccination history, etc.), and 3 ml of venous blood was collected from each participant by trained nurses in local clinics at least 30 days after the third dose of polio vaccine.

The study protocol was approved by the ethics committee of the Chongqing Center for Disease Control and Prevention (CDC) and all the procedures involved human beings were done in accordance with the Helsinki Declaration. Written informed consent was obtained from the legal guardians of participants before enrolment.

### Vaccines

According to the current routine polio primary vaccination procedure in China, participants received one dose of polio vaccine at 2, 3, and 4 months of age, respectively. The sIPV used in this study was manufactured by the Chinese Academy of Medical Sciences, which contained at least 30 D-Antigen units of poliovirus serotype 1, 32 D-Antigen units of poliovirus serotype 2, and 45 D-Antigen units of poliovirus serotype 3. The wIPV was manufactured by Sanofi Pasteur SA, France, which contained inactivated poliovirus serotype 1 (Mahoney strain; 40 D-Antigen units), poliovirus serotype 2 (MEF-1 strain; 8 D-Antigen units), and poliovirus serotype 3 (Saukett stain; 32 D-Antigen units). The bOPV used in this study was manufactured by the China National Pharmaceutical Group Corporation which contained at least 6.0 lgCCID_50_ per dose of poliovirus serotype 1 and 5.5 lgCCID_50_ per dose of poliovirus serotype 3.

### Laboratory testing

Blood Samples were immediately placed in transfer boxes and transported to the laboratory of the local CDC. The serum was separated and then stored in a refrigerator under − 20 °C. The samples were then transported to the polio laboratory of Chinese Academy of Medical Sciences for testing.

Micro neutralization assay was verified as gold standard by the Global Polio Laboratory Network [18], it was recommended by the WHO to measure the presence of type-specific neutralizing antibodies against Sabin-strain poliovirus serotype 1, 2, 3. Each serum sample was inactivated at 56 °C for 30 min before testing and then diluted from 1:8 to 1:1024 in two-fold serial dilutions. The production was then incubated in duplicate wells for 3 h at 36 °C with 50% tissue culture infective doses (TCID_50_) of poliovirus antigen. After incubation for 7 days, the highest dilution of serum that protected 50% of the cultures was recorded.

The endpoints were proportions of infants with seroprotection (sero-positivity), which is defined as original/neutralizing antibody titers of 8 or more to all three serotypes. When the titer of antibody was < 1:8, it was assigned a titer of 1:1, and when the titer was above the upper limit of detection (1/1024), it was assigned a titer of 1/2048. A serum sample with a titer of ≥ 1:8 for each poliovirus was considered to be positive [18]. Cell controls and a reference serum were included in each test to ensure the reproducibility of results.

### Statistical analysis

Statistical analysis was performed using SPSS 23.0 software. Median (M) and Quartile range (QR) were used to describe the distribution of neutralizing antibody titers. Fisher’s exact test was used to compare seropositivity among subgroups; Wilcoxon test and Kruskal-Wallis test were used to compare neutralizing antibody titers. *P* values less than 0.05 were considered to be statistically significant.

## Results

### Demographic information

A total of 528 individuals were enrolled in the study, of whom 469 met inclusion criteria, 458 participants completed the primary vaccination schedules, and 408 of them collected qualified blood specimens. The final cohort for immunogenicity test included 408 subjects: 154(37.75%) were in 3wIPV group, 66(16.18%) in 3sIPV group, 80 (19.61%) in 2wIPV + bOPV group and 108(26.47%) in 2sIPV + bOPV group. One hundred and nighty nine (199) mothers had a definite history of polio immunization, the vaccination schedules of their children were distributed as 137(87.26%) in 3wIPV group, 13(21.67%) in 3sIPV group, 44(55.00%) in 2wIPV + bOPV group and 5(4.63%) in 2sIPV + bOPV group (Table 1).

**Table 1:** Baseline characteristics of study participants (*N* = 408)

Subgroup	3wIPV (*n*/%)	3sIPV (*n*/%)	2wIPV + bOPV (*n*/%)	2sIPV + bOPV (*n*/%)
Sex				
Male	70(44.59)	32(53.33)	37(46.25)	63(58.33)
Female	84(55.41)	34(46.67)	43(53.75)	45(41.47)
Maternal immunization history				
Yes	137(87.26)	13(21.67)	44(55.00)	5(4.63)
No/ Unknown	17(12.74)	53(78.33)	36(45.00)	103(95.37)
Total	154	66	80	108

Note: N, number of participants; n (%), number (percentage) of participants in each group

### Immunogenicity of different polio primary immunization schedules

The neutralizing antibodies (NA) against the three poliovirus types and the seropositivity rates were measured within 1–6 months after the third dose. Our study showed that both current polio immunization series (3IPV & 2IPV + bOPV) induced high NA seropositivity rates against serotypes 1, 2, and 3 (99.75%, 99.02%, and 99.51%, respectively). The IPV-only series had 100% seropositivity rates against serotypes 1, 2, and 3. The sequential series of 2IPV + bOPV had 99% seropositivity rates against serotypes 1 and 3, and 98% against serotype 2(Table 2). The total median antibody titer of serotypes 1, 2, and 3 were 256, 96, and 256, respectively. The median antibody titer of the 2IPV + bOPV group was 2048 against serotype 1, 96 against serotype 2 and 2048 against serotype 3. The median antibody titer of the 3IPV group was 128 against serotype 1, 192 against serotype 2 and 256 against serotype 3(Table 2). Infants who received the primary schedule with one dose of bOPV had significantly higher antibody titers against serotypes 1 and 3 than did the schedule with only IPVs (*p* < 0.05). However, the primary series with 3IPV had higher antibody titer against serotype 2 than that with one bOPV (*p* < 0.05).

**Table 2:** Sero-positive rates and median titers of NAs against poliovirus serotype 1, 2, and 3 after the primary series vaccination

Group	Serotype 1					Serotype 2					Serotype 3				
	n(%)	*95%CI*	P*	M(IQR)	*P*	n(%)	*95%CI*	P*	M(IQR)	*P*	n(%)	*95%CI*	P*	M(IQR)	*P*
2IPV + bOPV(*n* = 188)	187(99.47)	97.05–99.91	0.46	2048(768–2048)	< 0.01	184(97.87)	94.65–99.17	0.04	96(64–192)	< 0.01	187(99.47)	97.05–99.91	1	2048(512–2048)	< 0.01
3IPV(*n* = 220)	220(100)	98.28–100		128(96–384)		220(100)	98.28–100		192(96–384)		219(99.55)	97.48–99.92		256(128–512)	

Note: * Fisher’s exact test for seropositive rates. M: median; IQR: inter quartile range; *P* values for the medians were calculated with Wilcoxon rank sum test

### Immunogenicity of immunization schedules with different strains

The overall seropositivity rates of 408 subjects in four groups ranged from 98.75 to 100% for serotype 1, from 96.25 to 100% for serotype 2, and from 98.75 to 100% for serotype 3. There was no significant difference in sero-positivities against serotype 1 and type 3 among groups. The seropositivity rate of serotype 2 was 96.25% in the 2wIPV + bOPV group, which was significantly lower than other groups (Table 3).

**Table 3:** Sero-protection of poliovirus against serotype 1, 2, and 3 by schedules with different strains

Group	n	Serotype 1			Serotype 2			Serotype 3		
		n(%)	95%CI	P*	n(%)	95%CI	P*	n(%)	95%CI	P*
3wIPV	154	154(100)	97.57–100	0.36	154(100)	97.57–100	0.042	153(99.35)	96.41–99.89	0.80
3sIPV	66	66(100)	94.50–100		66(100)	94.50–100		66(100)	94.50–100	
2wIPV + bOPV	80	79(98.75)	93.25–99.78		77(96.25)	89.55–98.72		79(98.75)	93.25–99.78	
2sIPV + bOPV	108	108(100)	96.57–100		107(99.07)	94.93–99.84		108(100)	96.57–100	

Note: * Fisher’s exact test

The median antibody titers in different groups against serotype 1 ranged from 96 (3wIPV group) to 2048 (2sIPV + bOPV and 2wIPV + bOPV groups), from 96 (2wIPV + bOPV group) to 384 (3sIPV group) against serotype 2, and ranged from 192(3wIPV group) to 2048(2wIPV + bOPV group) against serotype 3. There was no significant difference in antibody titer against serotype 3 between the 2wIPV + bOPV and the 2sIPV + bOPV groups (*p* > 0.05) (Tables 4 and 5). Both the groups of 2wIPV + bOPV and 2sIPV + bOPV had significantly higher antibody titers against serotypes 3 than other groups (*p* < 0.001). The highest antibody titers against serotype 2 were in the 3sIPV group (*p* < 0.001), followed by the 3wIPV group, the 2sIPV + bOPV group, and the 2wIPV + bOPV group.

**Table 4:** The neutralizing antibodies (NAs) against poliovirus serotype 1, 2, and 3 by schedules with different strains

Groups	Serotype 1		Serotype 2		Serotype 3	
	M	*P*< 0.01	M	*P*< 0.01	M	*P*< 0.01
3wIPV	96(64–192)		128(64–256)		192(96–384)	
3sIPV	512(256–1024)		384(192–832)		384(192–768)	
2wIPV + bOPV	2048(512–2048)		96(48–96)		2048(1024–2048)	
2sIPV + bOPV	2048(1024–2048)		128(96–192)		1024(416–2048)	

Note: M: median; IQR: inter quartile range; *P* values were calculated with Kruskal-Wallis test

**Table 5:** Multiple comparison of neutralizing antibodies (NAs) against poliovirus serotype 1, 2, and 3 by schedules with different strains

Groups	Serotype 1			Serotype 2			Serotype 3		
	3sIPV	2wIPV + bOPV	2sIPV + bOPV	3sIPV	2wIPV + bOPV	2sIPV + bOPV	3sIPV	2wIPV + bOPV	2sIPV + bOPV
3wIPV	< 0.001	< 0.001	< 0.001	< 0.001	< 0.001	1	< 0.001	< 0.001	< 0.001
3sIPV		0.072	< 0.001		< 0.001	< 0.001		< 0.001	< 0.001
2wIPV + bOPV			0.067			< 0.001			0.467

Note: Multiple comparison used Bonferroni method for *P* value adjustment, *P* < 0.0083 is considered to be significant

### Immunogenicity of immunization after different intervals

**Fig. 1:**
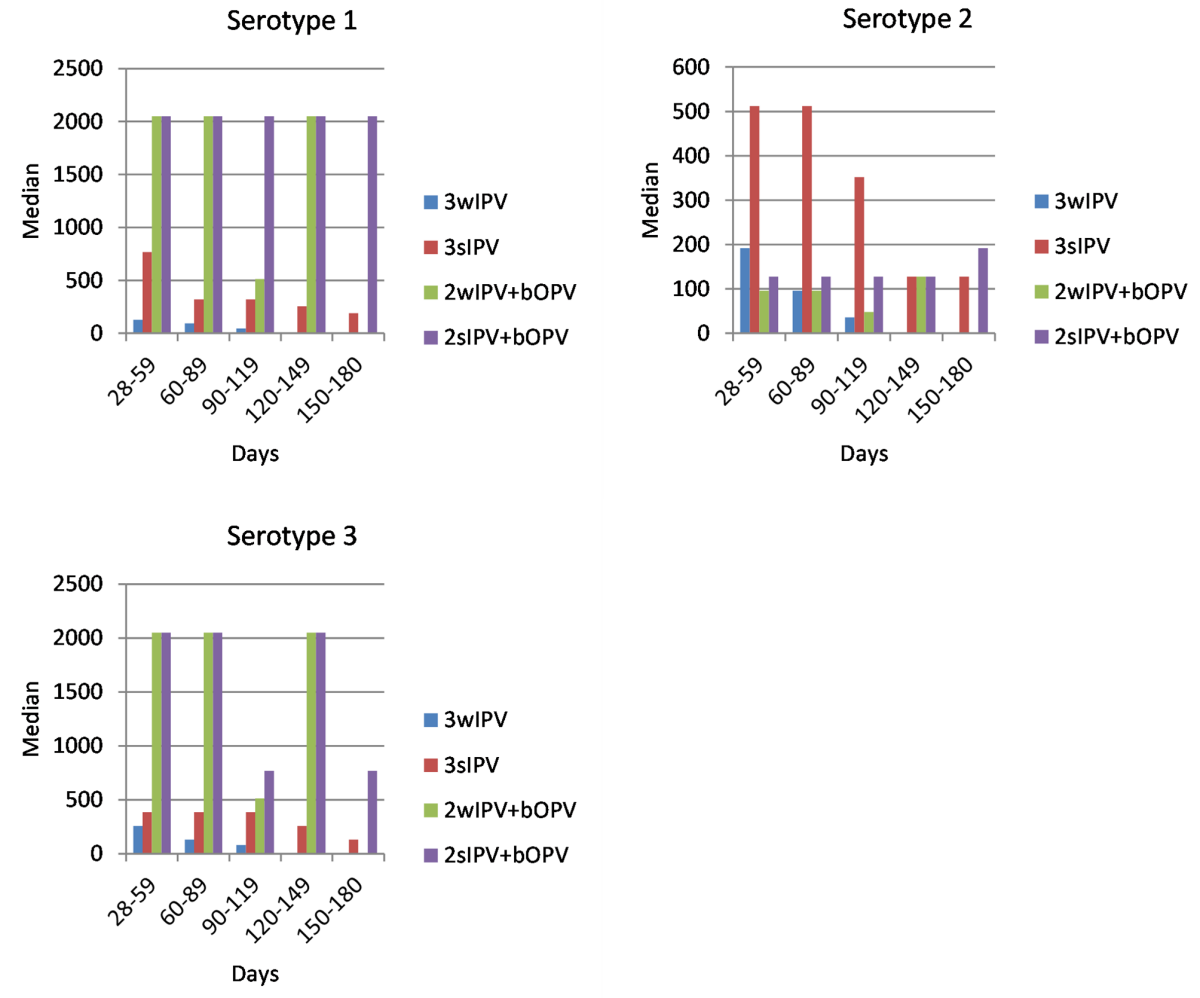
Median titers of antibody against type 1, 2, and 3 poliovirus after different vaccination schedules. wIPV: Salk strain inactivated polio vaccine; sIPV: Sabin strain inactivated polio vaccine; bOPV: bivalent oral polio vaccine; Polio serum antibody levels against the tree serotypes according to different time intervals between blood collection and the last dose of vaccination with 3wIPV, 3sIPV, 2wIPV + bOPV, and 2sIPV + bOPV schedules

**Fig. 2:**
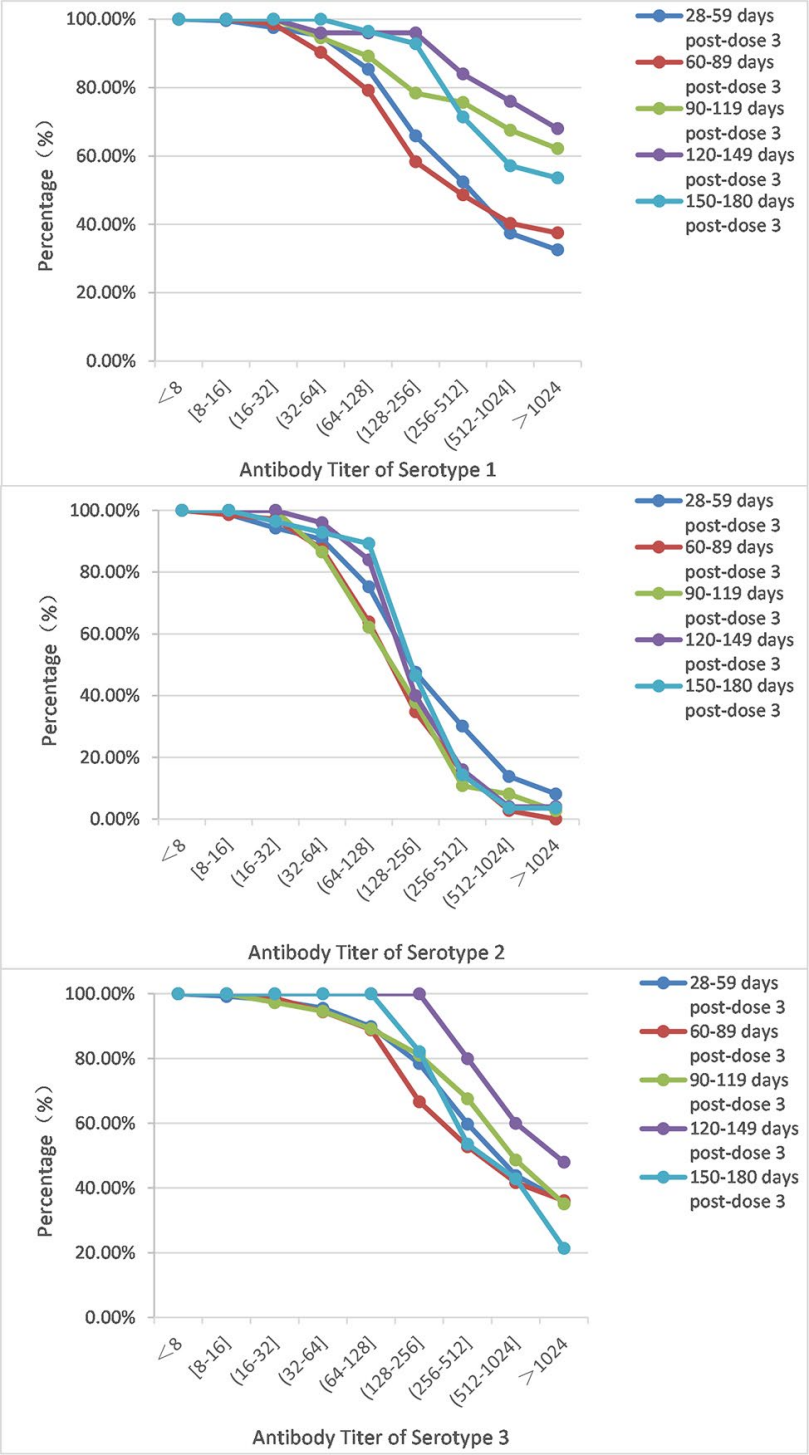
Reverse accumulated poliovirus antibody distribution curves for serotype 1, 2, and 3 at 28–180 days post the third vaccination

We further analyzed polio serum antibody levels of the three serotypes according to the time intervals between blood collection and the last dose to observe antibody maintenance levels. More than half of the subjects (60.29%, 246/408) were collected blood samples 28–59 days after the third dose of vaccination, followed with 17.65% (72/408), 9.07% (37/408), 6.13% (25/408), and 6.86% (28/408) at 60–89 days, 90–119 days, 120–149 days, and 150–180 days intervals, respectively. After three doses of primary immunization, we found the series sequenced with 1 dose of bOPV induced a stronger immune response to serotypes 1 and 3, with antibody titers being stable around 2000 from 28 to 180 days after the third vaccination compared with other IPV-only series. The median titer against serotype 3 in 2sIPV + bOPVgroup was around 2000 and was significantly more stable than other groups. For serotype 2, the median titer in the 3sIPV group was around 300 to 500, which was almost two to three times higher than the other groups in days 28–119 after the third vaccination, but it was only about 100 in days 120–180. (Fig. 1). Accumulated poliovirus antibody distribution for serotype 2 declined sharply over the titer 1: 128 and higher ones in all the intervals post the third vaccination (Fig. 2).

## Discussions

Our study showed that the current two polio primary vaccination schedules in China (IPV-IPV-IPV&IPV-IPV-bOPV) were highly immunogenic against poliovirus types 1, 2, and 3. Both primary immunization schedules produced a seropositivity rate of more than 96% for serotypes 1, 2, and 3, respectively, and there was statistical difference between the two schedules. The schedule of IPV-IPV-bOPV induced significantly higher levels of titers against serotypes 1 and 3 than the IPV-only schedule. The results implied that the primary sequential schedule containing only one bOPV could induce high levels of polio antibody type 1 and 3. While the primary schedule of IPV-IPV-IPV induced significantly higher levels of titers against serotype 2 than the IPV-IPV-bOPV, probably because it included an additional dose of vaccine containing the polio antigen type 2 components. The primary immunization with three doses of IPV was more immunogenic against type 2 poliovirus to induce adequate type 2 protection. Because the fourth dose of vaccine in the full course of polio immunization in China is a bOPV booster, it appears that the optimal immunization strategy is two doses of IPV primary vaccine plus a bOPV booster considering both economic and social benefits in the current situation. Though routine immunization schedules were different in countries [19–23], many countries chose sequential use of IPV and OPV.

The three doses of sIPV induced significantly stronger immune responses than the three doses of wIPV against serotypes 1, 2, and 3. However, this serological assay was based on the Sabin strain, so the results were friendlier to the Sabin strain of the vaccine. Among the different strain vaccines sequenced in the vaccinations, we found that the wIPV or sIPV sequenced with bOPV further enhanced immunity against serotypes 1, and 3 than w/sIPV-only schedules. This implied that attenuated polio vaccine for sequential polio vaccination could induce a more intense immune response to serotypes 1 and 3.

We have observed that serum antibodies to types 1, 2, and 3 produced by sequential vaccination with 2sIPV + bOPV were more stable than with 2wIPV + bOPV up to 6 months after primary immunization. It was obvious to find out that 3sIPV maintained a consistently high level of polio antibodies against type 1, 2, and 3 for 6 months compared to 3wIPV after immunizations. The median titers in the primary series with 1 sequential bOPV were observed to be significantly higher and more stable across 6 months than IPV-only series against types 1 and 3. This might be attributed to the mucosal immune mechanism produced by bOPV or some degree of cross-protection (heterotypic cross-reaction) by different immune ways. Therefore, further studies are needed to explore the immune mechanism of sequential inoculation with IPV & bOPV and the trend of antibody maintenance.

This study also has limitations. Firstly, we did not collect blood samples before the first poliovirus vaccine to assess maternal poliovirus neutralizing antibodies. Though interference of maternal antibodies with infant immune responses to polio vaccination appeared to be one potential barrier to children’s polio seroconversion rates after vaccination [24–27], this effect could be minimized by giving three doses of polio vaccines during the first year of life with optimal intervals. Secondly, the sample size was different among the four groups because of the convenient sampling method. Though we intended to enroll all the infants who came for their first polio vaccination in the vaccination clinic in the local women and children’s hospital, the sample size was unbalanced across groups due to the vaccine supply and impact of the COVID-19 epidemic. Thirdly, the 2sIPV + bOPV group has induced stronger immunity than 2wIPV + bOPV against all three polio serotypes, which can partly be attributed to the serological assay method based on the Sabin strain [28, 29].

## Conclusions

In summary, our findings showed that primary immunization with three doses of IPV induced higher levels of type 2 polio antibodies, and a sequential immunization schedule of only 1 dose of bOPV with multiple doses of IPVs could lead to further increase in antibody levels to serotypes 1 and 3. Therefore, under the threat of the current regional polio epidemic, we recommend that further polio immunization schedule adjustments in China should at least contain 1 dose of bOPV. Three doses of primary immunization using sIPV will provide a more sustained and high level of immune protection against polio.

## Data Availability

The datasets used and/or analyzed during the current study are available from the corresponding author upon reasonable request.

## Abbreviations

WPV: wild poliovirus
tOPV: trivalent oral attenuated polio vaccine
bOPV: bivalent oral poliovirus vaccine
IPV: inactivated polio vaccine
cVDPV: circulating vaccine-derived poliovirus
wIPV: Salk strain IPV
sIPV: Sabin strain IPV
CDC: Center for Disease Control and Prevention
QR: Quartile range
NA: neutralizing antibodies

## References

[1] Estivariz CF et al. Chapter 18: Poliomyelitis. Epidemiology and prevention of vaccine-preventable diseases, a.k.a. The Pink Book[M]. 14. Centers for Disease Control and Prevention, United States of America, 2021.

[2] WHO. Poliomyelitis (polio)[EB/OL]. https://www.who.int/health-topics/poliomyelitis#tab=tab_1.

[3] Global Polio Eradication Initiative. Polio Eradication and Endgame Strategic Plan 2013–2018[EB/OL]. https://polioeradication.org/who-we-are/strategic-plan-2013-2018/.

[4] Agency Of Disease Control And Prevention. Vaccination work specifications[EB/OL]. (2016-12-29)http://www.nhc.gov.cn/cms-search/xxgk/getManuscriptXxgk.htm?id=8033406a995d460f894cb4c0331cb400.

[5] WHO (2022). Polio vaccines: WHO position paper – June 2022[J]. Wkly Epidemiol Rec.

[6] Macklin GR, O’Reilly KM, Grassly NC (2020). Evolving epidemiology of poliovirus serotype 2 following withdrawal of the serotype 2 oral poliovirus vaccine[J]. Science.

[7] Cooper LV, Bandyopadhyay AS, Gumede N (2022). Risk factors for the spread of vaccine-derived type 2 polioviruses after global withdrawal of trivalent oral poliovirus vaccine and the effects of outbreak responses with monovalent vaccine: a retrospective analysis of surveillance data for 51 countries in Africa[J]. Lancet Infect Dis.

[8] Kalkowska DA, Pallansch MA, Wilkinson A (2021). Updated characterization of outbreak response strategies for 2019–2029: impacts of using a novel type 2 oral Poliovirus Vaccine Strain[J]. Risk Anal.

[9] Blake IM, Pons-Salort M, Molodecky NA (2018). Type 2 Poliovirus Detection after global withdrawal of trivalent oral Vaccine[J]. N Engl J Med.

[10] Saleem AF, Yousafzai MT, Mach O (2018). Evaluation of vaccine derived poliovirus type 2 outbreak response options: a randomized controlled trial, Karachi, Pakistan[J]. Vaccine.

[11] Alleman MM, Jorba J, Greene SA (2020). Update on vaccine-derived Poliovirus outbreaks - Worldwide, July 2019-February 2020[J]. MMWR Morb Mortal Wkly Rep.

[12] Bigouette JP, Henderson E, Mohamed A, Traoré (2023). Update on vaccine-derived Poliovirus outbreaks - Worldwide, January 2021-December 2022[J]. MMWR Morb Mortal Wkly Rep.

[13] Stehling-Ariza T, Wilkinson AL, Diop OM (2023). Surveillance to Track Progress toward Poliomyelitis Eradication-Worldwide, 2021–2022. Morb Mortal Wkly Rep.

[14] Chen Qiang Q, Yuhua L, Hongying (2021). Compare the polio antibody level before and after the conversion of inactivated poliovirus vaccine immunization program[J]. Chin J Microbiol Immunol.

[15] Yang Xiuhui Z, Yong Z, Shuangli (2021). To evaluate the neutralizing antibody of poliovirus in children aged < 12 years of Fujian Province[J]. Chin J Experimental Clin Virol.

[16] Wang Qing Z, Yuanyuan X (2019). Immunogenicity of sequential polio vaccination schedules that use inactivated polio vaccine as the first dose[J]. Chin J Vaccines Immun.

[17] Wen Ning S, Qiru A, Zhijie (2018). Considerations and suggestions for polio vaccination strategies in China[J]. Chin J Vaccines Immunizations.

[18] Weldon WC, Oberste MS, Pallansch MA (2016). Standardized methods for detection of Poliovirus Antibodies[J]. Methods Mol Biol.

[19] Parent DC, I, Merchant AT, Fisher-Hoch S (2003). Serological response and poliovirus excretion following different combined oral and inactivated poliovirus vaccines immunization schedules[J]. Vaccine.

[20] Resik S, Tejeda A, Sutter RW (2013). Priming after a fractional dose of inactivated poliovirus vaccine[J]. N Engl J Med.

[21] Geneva: World Health Organization. Considerations for the timing of a single dose of IPV in the routine immunization schedule[EB/OL]. (2014-06-03)https://www.who.int/publications/i/item/WER8901.

[22] Alexander LN, Seward JF, Santibanez TA (2004). Vaccine policy changes and epidemiology of Poliomyelitis in the United States[J]. JAMA.

[23] Jafari H, Deshpande JM, Sutter RW (2014). Polio eradication. Efficacy of inactivated poliovirus vaccine in India[J]. Science.

[24] Gaensbauer JT, Gast C, Bandyopadhyay AS (2018). Impact of maternal antibody on the immunogenicity of inactivated Polio Vaccine in infants immunized with bivalent oral Polio Vaccine: implications for the Polio Eradication Endgame[J]. Clin Infect Dis.

[25] Asturias EJ, Bandyopadhyay AS, Self S (2016). Humoral and intestinal immunity induced by new schedules of bivalent oral poliovirus vaccine and one or two doses of inactivated poliovirus vaccine in latin American infants: an open-label randomised controlled trial[J]. Lancet.

[26] Li-na GENG, Kai CHU, Guang-wei SUN (2017). Effectiveness of OPV and the influence of maternal anti-poliovirus antibodies on evaluation of OPV in the Chinese infants[J]. Mod Prev Med.

[27] Min CUI, De-yu JIANG, Qing HE (2016). Changes in poliovirus neutralizing antibodies among puerperas and infants: a longitudinal study[J]. Chin J Public Health.

[28] Albrecht P, Enterline JC, Boone EJ (1983). Poliovirus and Polio antibody assay in HEp-2 and Vero cell cultures[J]. J Biol Stand.

[29] Xu J, Wang Q, Kuang S (2021). Immunogenicity of sequential poliovirus vaccination schedules with different strains of Poliomyelitis vaccines in Chongqing, China: a cross-sectional survey[J]. Hum Vaccin Immunother.

